# Epidemiologic trends and mortality in nontuberculous mycobacterial lung disease: a nationwide cohort study in Taiwan (2011–2020)

**DOI:** 10.7189/jogh.16.04292

**Published:** 2026-09-11

**Authors:** Chih-Hao Chang, Yu-Feng Wei, Ping-Huai Wang, Sheng-Wei Pan, Su-Mei Wang, Chin-Hao Chang, Chin-Chung Shu

**Affiliations:** 1 Division of Pulmonary and Critical Care Medicine, Department of Internal Medicine, Jen Ai Hospital, Taichung, Taiwan; 2 Division of Pulmonary and Critical Care Medicine, Department of Internal Medicine, New Taipei City Municipal Tucheng Hospital, New Taipei City, Taiwan; 3 Department of Thoracic Medicine, Chang Gung Memorial Hospital, Linkou, Taoyuan City, Taiwan; 4 Department of Internal Medicine, E-Da Cancer Hospital, Kaohsiung, Taiwan; 5 School of Medicine for International Students, College of Medicine, I-Shou University, Kaohsiung, Taiwan; 6 Division of Thoracic Medicine, Far Eastern Memorial Hospital, New Taipei City, Taiwan; 7 Department of Nursing, Asia Eastern University of Science and Technology, New Taipei City, Taiwan; 8 Department of Chest Medicine, Taipei Veterans General Hospital, Taipei, Taiwan; 9 School of Medicine, National Yang Ming Chiao Tung University, Taipei, Taiwan; 10 Department of Medical Research, National Taiwan University Hospital, Taipei, Taiwan; 11 Department of Internal Medicine, National Taiwan University Hospital, Taipei, Taiwan; 12 Graduate Institute of Clinical Medicine, National Taiwan University College of Medicine, Taipei, Taiwan

**Keywords:** lung disease, nontuberculous mycobacteria, treatment, incidence, NTM

## Abstract

**Background:**

Nontuberculous mycobacterial lung disease (NTM-LD), a condition characterised by a heterogeneous course and complex multidrug treatment, is becoming an increasingly prevalent global health concern. Given that population-based data on real-world management and outcomes are limited, we aimed to characterise nationwide trends in treatment initiation, real-world treatment courses, and cause-specific mortality among patients treated for NTM-LD, as well as identify factors associated with three-year mortality.

**Methods:**

In this retrospective cohort study, we identified patients in Taiwan’s National Health Insurance Research Database who had been newly diagnosed with NTM-LD between July 2011 and December 2020 based on diagnostic ICD codes. All identified cases served as the numerator for incidence estimation; the treated NTM-LD cohort comprised patients initiating a multidrug anti-NTM regimen sustained for at least 28 days. The primary outcome was three-year all-cause mortality, ascertained through the national death registry. We used multivariable Cox proportional hazards regression to estimate adjusted hazard ratios and 95% confidence intervals for mortality risk factors.

**Results:**

Among 15,589 patients diagnosed with NTM-LD, 3,762 received anti-NTM treatment and comprised the treated NTM-LD cohort. The incidence of NTM-LD increased from 5.4 to 8.9 per 100,000 person-years between 2012 and 2019. The median treatment duration was 158 days. The three-year mortality rate was 19.4%, with cancer, chronic airway diseases, and pneumonia being the leading causes of death. Being aged ≥65 years; male; having chronic respiratory failure, cancer, heart failure, chronic obstructive pulmonary disease, or idiopathic pulmonary fibrosis; being hospitalised (the strongest factor, adjusted hazard ratio = 3.70), and receiving intravenous antibiotic treatment were independently associated with three-year mortality.

**Conclusions:**

Our findings point to an increasing burden of treated NTM-LD in Taiwan and a substantial three-year mortality rate. Older age and multiple comorbidities were independently associated with mortality. These findings should be interpreted with caution because cases were defined without microbiologic or radiographic confirmation, and untreated individuals were excluded.

Nontuberculous mycobacterial lung disease (NTM-LD) is a chronic pulmonary infection caused by environmental mycobacteria [1,2]. Its global incidence and prevalence have risen significantly over the past two decades, particularly among older adults, individuals with underlying structural lung disease, and immunocompromised populations [3,4]. The condition contributes to progressive lung function decline and has been associated with increased morbidity and mortality [5], with a long-term follow-up study reporting it to have a 15-year cumulative mortality rate of 36.4% [6].

Despite growing awareness of its importance, clinical management of NTM-LD remains highly challenging [7], with clinical course and prognosis being variable and influenced by factors such as the specific NTM species involved [8]. Standard regimens are lengthy; they typically continue for at least 12 months after sputum culture conversion and require multidrug combinations with potentially significant adverse effects [9]. This frequently leads to poor adherence, early discontinuation, and treatment failure [10]. Moreover, regimens are often empirically tailored based on species identification, drug susceptibility testing, and individual patient tolerance, contributing to substantial variability in real-world prescribing patterns [11].

A better understanding of NTM-LD thus requires long-term population-based surveillance [12,13]. In addition, the condition predominantly affects older adults with multiple comorbidities, and treatment typically requires prolonged multidrug regimens that are often associated with substantial toxicity and limited tolerability, making treatment initiation challenging and resulting in deviations from guideline recommendations in real-world prescription practices [12]. Despite the growing clinical importance of NTM-LD, population-based data on treatment patterns, mortality, and prognostic factors remain limited, particularly in Asia [14].

To our knowledge, nationwide population-based data on the epidemiology, treatment patterns, and mortality of NTM-LD in Taiwan remain limited. We therefore conducted a nationwide cohort study using Taiwan’s National Health Insurance Research Database (NHIRD) to investigate incidence trends, real-world treatment patterns, mortality, and associated risk factors. Specifically, we aimed to describe nationwide trends among patients treated for NTM-LD in treatment initiation and incidence between 2011 and 2020, characterise real-world treatment courses and regimen heterogeneity, quantify three-year all-cause and cause-specific mortality, and identify clinical factors independently associated with three-year mortality.

## METHODS

We obtained our data from the NHIRD, which is derived from Taiwan’s National Health Insurance programme and covers >99% of residents. The NHIRD is managed by the Health and Welfare Data Science Center, Ministry of Health and Welfare. We report our findings per the STROBE guidelines for cohort studies (Checklist S1 in the **Online Supplementary Document**).

**Figure 1:**
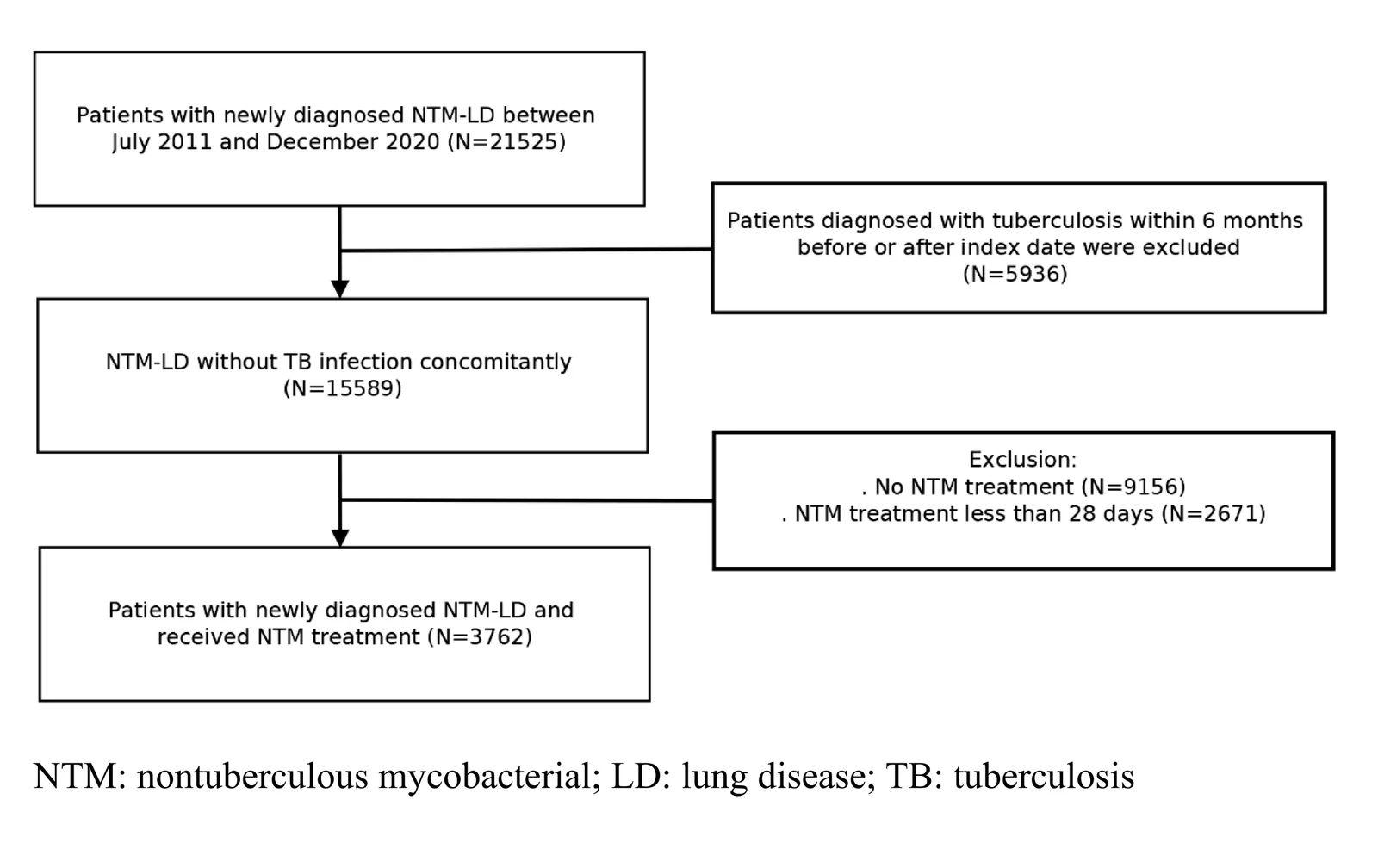
Study population flow diagram. NTM-LD – nontuberculous mycobacterial lung disease, TB – tuberculosis.

We identified patients diagnosed with NTM-LD between July 2011 and December 2020 based on ICD-9-CM codes 031.0, 031.2, 031.8, and 031.9, and ICD-10-CM codes A31.0, A31.2, A31.8, and A31.9. We included patients with at least two outpatient or emergency-department visits with NTM infection among the three primary diagnoses, or at least one hospitalisation with NTM infection among the five primary diagnoses. We excluded patients with pulmonary tuberculosis within six months before or after the NTM diagnosis date (the index date) and those who did not receive NTM treatment or had received treatment for less than 28 days (**Figure 1**).

Because microbiologic species identification and drug-susceptibility results are not available in the NHIRD, an anti-NTM regimen was operationally defined as the concurrent prescription of at least two antimycobacterial agents appropriate for the treatment of NTM-LD: a macrolide (azithromycin or clarithromycin) combined with one or more of ethambutol, a rifamycin (rifampicin or rifabutin), a fluoroquinolone, amikacin, or another agent recommended by the relevant guidelines [10,15]. We regarded a change in drug combination during continuous therapy as part of the same treatment course if no interruption exceeded 28 days; we counted a new course only when therapy was reinitiated after a gap longer than 28 days. We defined treatment with intravenous antibiotics as the administration of any parenteral antimycobacterial agent (most commonly intravenous amikacin) at any point. This claims-based algorithm relies on diagnostic and prescription codes, rather than the combined microbiologic, radiographic, and clinical criteria specified by the relevant guideline [10].

### Main outcome and covariates

The primary outcome was three-year all-cause mortality, with the cause of death data obtained from Taiwan’s Ministry of Health and Welfare death registry. We otherwise defined a treatment course as continuous regimen for NTM-LD lasting >28 days without a treatment gap exceeding 28 days.

We selected covariates *a priori* based on their clinical and biological plausibility and their established prognostic relevance in NTM-LD [16,17], rather than based on statistical significance. They comprised demographic factors (age, sex), care setting, pulmonary and extrapulmonary comorbidities (identified using ICD codes and catastrophic illness registrations in the NHIRD), and treatment-related characteristics (treatment duration, number of treatment courses, interval to treatment, and use of intravenous antibiotics). We could not incorporate several otherwise recognised prognostic variables, including body-mass index, smoking status, microbiologic burden, and frailty, as they were not recorded in the NHIRD.

### Statistical analysis

We compared categorical variables using chi-squared tests and continuous variables using Student’s *t*-tests or analysis of variance (for comparisons involving multiple groups). We employed Cox proportional hazards regression to calculate multivariable adjusted hazard ratios (aHRs) and 95% confidence intervals (CIs) for factors associated with outcomes. Survival time was measured from the date of treatment initiation (the cohort entry point) to death or the end of the three-year follow-up window, with administrative censoring at the database cut-off of 31 December 2020. By defining cohort entry at treatment initiation, rather than at diagnosis, we sought to reduce immortal-time bias arising from the interval between diagnosis and the start of therapy. For patients with more than one treatment course, we summed the total treatment duration across courses and modelled the number of courses as a separate covariate. We evaluated the proportional hazards assumption using scaled Schoenfeld residuals and observed no significant violation.

We performed all statistical analyses using SAS, version 9.4 (SAS Institute, Cary, North Carolina, USA). A two-sided *P*-value <0.05 indicated statistical significance.

## RESULTS

**Figure 2:**
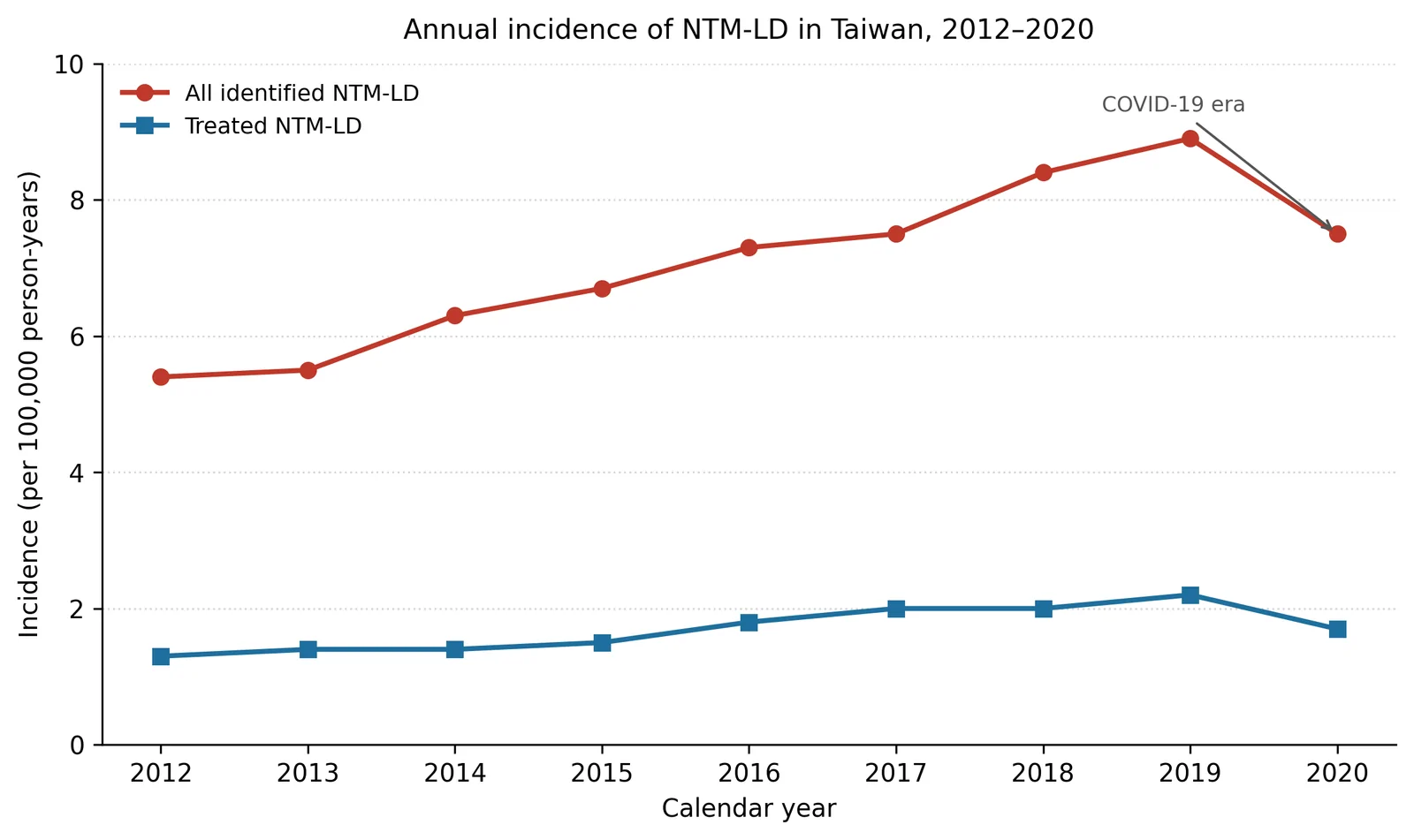
Annual incidence of all identified NTM-LD and of treated NTM-LD in Taiwan, 2012–2020 (per 100,000 person-years). The apparent decline in 2020 coincides with the COVID-19 pandemic. Data are derived from **Table 1**; 2011 is omitted because it represents only six months of observation. NTM-LD – nontuberculous mycobacterial lung disease.

A total of 21,525 patients were diagnosed with NTM-LD between July 2011 and December 2020 (**Figure 1**). We excluded 5,936 for having a diagnosis of tuberculosis within six months before or after the index date, leaving 15,589 eligible patients. Among them, 9,156 received no anti-NTM treatment and 2,671 received treatment for less than 28 days. All patients meeting the ICD-based case definition, *i.e.* identified as having NTM-LD (n = 15,589) contributed to the estimation of annual NTM-LD incidence, while all clinical, treatment, and mortality analyses were restricted to the treated NTM-LD cohort (n = 3,762). The number of patients treated for NTM-LD grew from 314 in 2012 to 525 in 2019; the incidence of NTM-LD in this period rose from 5.4 to 8.9 per 100,000 person-years, while that of treated NTM-LD increased from 1.3 to 2.2 per 100,000 person-years (**Figure 2**, **Table 1**).

**Table 1:** Annual incidence of nontuberculous mycobacterial lung disease (NTM-LD) in Taiwan, 2011–2020

Year	Beneficiaries	NTM-LD	NTM-LD with treatment	NTM-LD incidence rate*	Treated NTM-LD incidence rate per 100,000 person-years
2011*	23,198,664	482	111	N/A	N/A
2012	23,280,949	1,249	314	5.4	1.3
2013	23,462,863	1,288	326	5.5	1.4
2014	23,621,599	1,484	328	6.3	1.4
2015	23,737,221	1,593	359	6.7	1.5
2016	23,814,584	1,740	421	7.3	1.8
2017	23,880,332	1,798	485	7.5	2.0
2018	23,948,108	2,017	481	8.4	2.0
2019	24,020,428	2,148	525	8.9	2.2
2020	23,986,997	1,790	412	7.5	1.7

NTM-LD – nontuberculous mycobacterial lung disease

*Data only includes the period from July to December; therefore, the incidence rate for 2011 is not directly comparable to other full-calendar years.

### The characteristics of NTM-LD patients with treatment

The mean age of the treated cohort was 60.4 years (standard deviation (SD) = 15.3) and 50.4% were male (**Table 2**). Most patients (n = 2,402, 64.2%) received care at medical centres, while about a quarter (n = 1,004, 26.9%) were treated at regional hospitals. The most common comorbidities were chronic obstructive pulmonary disease (COPD) (24.9%) and bronchiectasis (23.1%).

**Table 2:** Clinical characteristics for NTM-LD with treatment and subgroups with survival and mortality outcomes in NHIRD*

	All (n = 3,762)	Mortality, three-year follow-up (n = 729)	Survival, three-year follow-up (n = 3,033)	*P*-value
**Age in years, x̄ (SD)**	60.4 (15.3)	68.4 (14.0)	58.4 (15.0)	0.014
**Age groups**				
≥65	1,585 (42.1)	471 (64.6)	1,114 (36.7)	<0.001
<65	2,177 (57.9)	258 (35.4)	1,919 (63.3)	
**Male sex**	1,894 (50.4)	463 (63.5)	1,431 (47.2)	<0.001
**Index year**				<0.001
2011–2015	1,438 (38.2)	334	1,104	
2016–2020	2,324 (61.8)	395	1,929	
2011†	111	32	79	
2012	314	74	240	
2013	326	79	247	
2014	328	81	247	
2015	359	68	291	
2016	421	93	328	
2017	485	100	385	
2018	481	95	386	
2019	525	79	446	
2020	412	28	384	
**Care setting**				<0.001
Medical center	2,402 (64.2)	429 (17.9)	1,973 (82.1)	
Regional hospital	1,004 (26.9)	238 (23.7)	766 (76.3)	
District hospital or lower	333 (8.9)	58 (17.4)	275 (82.6)	
**Comorbidities**				
Chronic respiratory failure	160 (4.3)	118 (16.2)	42 (1.4)	<0.001
Cancer	589 (15.7)	266 (36.5)	323 (10.7)	<0.001
Cirrhosis	8 (0.2)	5 (0.7)	3 (0.1)	0.002
End-stage renal disease	120 (3.2)	43 (5.9)	77 (2.5)	<0.001
Heart failure	196 (5.2)	89 (12.2)	107 (3.5)	<0.001
Diabetes mellitus	669 (17.8)	190 (26.1)	479 (15.8)	<0.001
Asthma	357 (9.5)	91 (12.5)	266 (8.8)	0.002
COPD	938 (24.9)	294 (40.3)	644 (21.2)	<0.001
Bronchiectasis	867 (23.1)	117 (16.1)	750 (24.7)	<0.001
Idiopathic pulmonary fibrosis	158 (4.2)	59 (8.1)	99 (3.3)	<0.001
Gastroesophageal reflux disease	517 (13.7)	110 (15.1)	407 (13.4)	0.240
Sinusitis	83 (2.2)	18 (2.5)	65 (2.1)	0.590
Post-transplant	44 (1.1)	16 (2.1)	28 (0.9)	0.004
Autoimmune disease‡	144 (3.8)	27 (3.7)	117 (3.9)	0.846
Sjögren syndrome	146 (3.9)	20 (2.7)	126 (4.2)	0.077
Follow-up in days, x̄ (SD)	786.1 (372.1)	404.7 (298.7)	877.7 (327.0)	0.002
Hospitalisation	1,593 (42.3)	562 (35.3)	1031 (64.7)	<0.001
Total treatment duration in days, x̄ (SD)	212.5 (187.4)	145.8 (137.2)	228.5 (194.2)	<0.001
First-course duration, days, x̄ (SD)	197.2 (174.0)	138.6 (129.8)	211.2 (180.2)	<0.001
Diagnosis-to-treatment interval, days, x̄ (SD)	57.1 (158.2)	53.0 (136.2)	58.1 (163.2)	<0.001
**Number of treatment courses**				<0.001
1 course	3,463 (92.1)	696 (95.5)	2,767 (91.2)	
2 courses	270 (7.2)	30 (4.1)	240 (8.6)	
>2 courses	29 (0.8)	3 (0.4)	26 (0.8)	
Treated with IV antibiotics	515 (13.7)	168 (23.0)	347 (12.3)	<0.001
Treated with IV amikacin	308 (8.2)	71 (9.7)	237 (7.8)	0.088

COPD – chronic obstructive pulmonary disease, IV – intravenous, NHIRD – National Health Insurance Research Database, NTM-LD – nontuberculous mycobacterial lung disease, SD – standard deviation, x̄ – mean

*Presented as n (%) unless specified otherwise.

†Represents only six months of data,

‡Autoimmune disease includes systemic lupus erythematosus, rheumatoid arthritis, dermatomyositis, and polymyositis.

### Treatment course and regimen

The median total treatment duration was 158 days, and the median interval between diagnosis and treatment initiation was 1 day. Of 3,762 patients, 3,463 (92.1%) underwent a single course (median duration of 147 days), 270 (7.2%) received two courses, and 29 (0.8%) received more than two. In addition, 515 patients (13.7%) initiated treatment with intravenous (IV) antibiotics and 308 (8.2%) received regimens containing IV amikacin (**Table 3**).

**Table 3:** Common initial treatment regimen (n = 3762)

Initial regimen*	n (%)
Macrolide + ethambutol + (rifabutin or rifampicin)	682 (18.1)
Macrolide + rifampicin + isoniazid + ethambutol	10 (0.3)
Macrolide + levofloxacin	413 (11.0)
Macrolide + rifampicin	58 (1.5)
Macrolide + rifabutin	18 (0.5)
Macrolide + ethambutol	112 (3.0)
Macrolide + rifampicin + isoniazid	29 (0.8)
Macrolide + doxycycline	94 (2.5)
Initial intravenous-based regimen	1,253 (33.3)
*IV amikacin plus another IV antibiotic*	400 (10.6)
*IV amikacin*	217 (5.8)
*Other IV antibiotic*	636 (16.9)
Miscellaneous	1,093 (29.1)

IV – intravenous

*Macrolide includes azithromycin or clarithromycin.

### Three-year mortality and the risk factors

The median follow-up was 1,053.5 days, while the three-year mortality rate among treated patients was 19.4%. The leading causes of death were cancer (n = 239, 32.8%), chronic airway diseases (n = 95, 13.9%), and pneumonia (n = 72, 9.9%). A total of 729 patients died during follow-up (**Table 2**).

**Figure 3:**
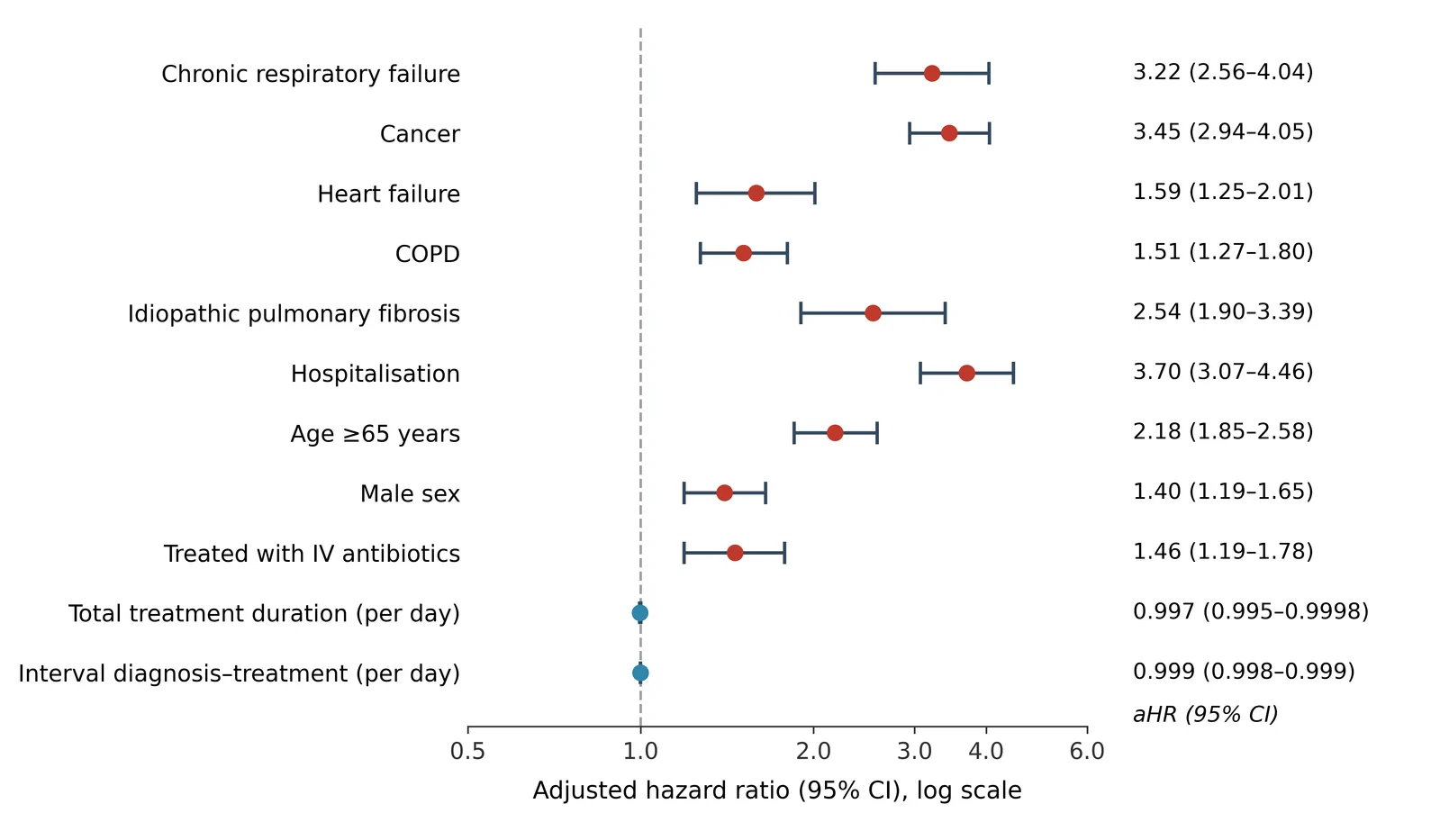
Forest plot of the multivariable Cox proportional hazards model for three-year all-cause mortality among treated patients with NTM-LD. Squares represent aHRs and horizontal lines represent 95% CIs; the dashed line denotes the null value (hazard ratio = 1). Estimates are derived from Table S1 in the **Online Supplementary Document**. aHR – adjusted hazard ratio, CI – confidence interval, COPD – chronic obstructive pulmonary disease, NTM-LD – nontuberculous mycobacterial lung disease.

In the univariable analysis, being aged ≥65 years, being male; having chronic respiratory failure, cancer, cirrhosis, end-stage renal disease, heart failure, diabetes mellitus, asthma, COPD, bronchiectasis, idiopathic pulmonary fibrosis (IPF), or post-transplant status; being hospitalised; and receiving treatment with IV antibiotics were associated with three-year mortality (Table S1 in the **Online Supplementary Document**). In multivariable analysis, being aged ≥65 years; being male; having chronic respiratory failure, cancer, heart failure, COPD, or IPF; being hospitalised, and receiving IV antibiotic treatment remained independently associated with higher mortality (**Figure 3**; Table S1 in the **Online Supplementary Document**).

A longer total treatment duration was associated with lower mortality (aHR = 0.997 per day; 95% CI = 0.995–0.9998), and the interval between diagnosis and treatment initiation showed a statistically significant, but clinically negligible association (aHR = 0.999 per day; 95% CI = 0.998–0.999).

The overall three-year mortality of 19.4% masked substantial heterogeneity across subgroups. Absolute mortality was markedly higher among patients aged ≥65 years than those aged <65 years (29.7% *vs*. 11.9%), and among men than women (24.4% *vs*. 14.2%). It was particularly high in clinically vulnerable subgroups, such as patients with chronic respiratory failure (73.8%), heart failure (45.4%), cancer (45.2%), IPF (37.3%), and COPD (31.3%) as well as among hospitalised patients (35.3%) and those treated with IV antibiotics (32.6%). By care setting, mortality was highest at regional hospitals (23.7%) and similar between medical centres (17.9%) and district hospitals (17.4%). Nationally, the number of patients treated for NTM-LD rose steadily across the decade (**Figure 2**), while long-term mortality was concentrated among older men and patients with cardiorespiratory and malignant comorbidity.

## DISCUSSION

To our knowledge, this is the first population-based study to evaluate the incidence of NTM-LD in Taiwan. Chen and colleagues previously reported on 558 patients with NTM infection using a cohort of 2 million representative individuals from Taiwan’s NHIRD [18]. In contrast, we used a comparatively larger nationwide dataset that allowed us to draw real-world insights into NTM-LD, including trends in incidence, treatment patterns, mortality, and associated risk factors. In our study, the incidence of identified NTM-LD increased from 5.4 to 8.9 per 100,000 person-years between 2012 and 2019. A study using health insurance claims data reported an increase in the prevalence of NTM-LD in Germany [19], while a nationwide retrospective cohort study in Denmark showed that the incidence of pulmonary NTM disease grew between 2000 and 2017 [20]. In Oregon, USA, a population-based study using state-wide mycobacterial laboratory data reported an increase in the incidence from 4.8 to 5.6 per 100,000 from 2007 to 2012 [21]. However, not all patients with NTM-LD receive treatment [10]. In our research, NTM-LD cases with anti-NTM treatment accounted for approximately one-fourth of all NTM-LD cases, with the incidence increasing from 1.3 to 2.2 per 100,000 population.

We observed a decline in the incidence of NTM-LD in 2020, decreasing to 7.5 per 100,000 person-years from a peak of 8.9 in 2019. This decrease may be related to the COVID-19 pandemic, whereby patient’s avoidance of attending healthcare facilities and the prioritisation of diagnostic resources for COVID-19 treatments likely delayed many diseases diagnosis [22,23]. Because our case definition required either repeated outpatient visits or hospitalisation, reduced healthcare utilisation during the pandemic may have resulted in fewer patients receiving an NTM-LD diagnosis.

A total of 9,156 (58.7%) eligible patients with NTM-LD in our source dataset did not receive anti-NTM antibiotic therapy. This pattern is not unique to Taiwan and has been reported in other studies, where a considerable proportion of patients are monitored, rather than immediately given antimicrobial treatment [24]. The decision to initiate therapy for NTM-LD is often complex, given the variable disease course and the potential toxicity associated with prolonged multidrug treatment. Jhun and colleagues analysed 1,445 patients with NTM-LD, of whom 893 (62%) initiated treatment, while 552 (38%) were monitored without anti-NTM treatment [6]. A real-world study in Germany showed that the mean treatment duration for patients managed by general practitioners was shorter than for those treated by pulmonologists (113 *vs*. 241 days) [25]. Similarly, a study from the Netherlands found the average treatment duration to be about four months [11]. In the study, initial treatment regimens for NTM-LD revealed an inconsistency between real-world clinical practice and international guideline recommendations. The standard triple therapy recommended by ATS/IDSA – consisting of a macrolide, ethambutol, and rifampicin [10] – was used in only 18% of patients; conversely, 695 patients (18.5%) received dual therapy, while 29.1% were classified as receiving miscellaneous regimens. Limited adherence to guideline-recommended therapy may reflect clinicians’ concerns regarding adverse effects or diagnostic uncertainty during early disease management [26]. A recent study from a tertiary referral centre reported that NTM-LD treatment is frequently complicated by adverse events [27]. Another study reported that 43% of patients receiving treatment for NTM-LD experienced adverse drug reactions [28]. Such heterogeneous prescribing patterns raise concerns about the potential development of macrolide resistance [29].

In our study, most patients received care at medical centres or regional hospitals, reflecting the complexity of NTM-LD management and the specialised resources required for diagnosis and treatment. The median treatment duration was only 158 days (mean 212.5 days, standard deviation = 187.4), shorter than the prolonged treatment courses recommended by current international guidelines [10]. First, treatment discontinuation due to adverse drug reactions and poor tolerability is common, with previous studies reporting adverse events in approximately 40% of treated patients [30]. Second, competing mortality in this older and highly comorbid population within our study may have limited the observed treatment duration. Third, diagnostic uncertainty and the absence of microbiologic data in claims databases [14] may have resulted in treatment discontinuation when the diagnosis was reconsidered or may have captured empirical treatment courses not intended as definitive therapy. Collectively, these findings suggest that the relatively short treatment duration observed in this study reflects real-world treatment challenges. Marras *et al.* reported that NTM-LD increased the risk of all-cause mortality (HR = 2.06; 95% CI = 1.52–2.79, *P* < 0.001) compared to the control group [31]. Risk factors such as being older, being male, and having a low body mass index have been consistently associated with poorer survival outcomes in patients with NTM-LD [32]. In our nationwide study, the leading causes of death in patients with NTM-LD were cancer, chronic airway diseases, and pneumonia. These findings are consistent with a previous study in one tertiary-care hospital [33].

We found that being male was a risk factor for three-year mortality from NTM-LD, which aligns with the findings of previous cohorts from Taiwan and South Korea [16,33,34]. The mechanisms underlying this association, however, remain unclear. We further found an association between longer total treatment duration and reduced three-year mortality in patients with NTM-LD. Although the magnitude of the daily risk reduction was modest, the cumulative survival benefit may be clinically meaningful given the prolonged treatment courses typically required for NTM-LD. A previous study using the National Taiwan University Hospital–Integrative Medical Database showed that ≥91 days of anti-NTM treatment within three consecutive months was associated with reduced three-year mortality [35]. However, the total duration of treatment in our study was more strongly associated with survival than the duration of the first treatment course in the study.

Simultaneously, we identified the interval between diagnosis by disease coding and treatment initiation as a statistically significant factor negatively associated with three-year mortality in patients with NTM-LD (aHR = 0.999; 95% CI = 0.998–0.999, *P* < 0.001). The observed inverse hazard ratio may reflect patients with a longer interval before initiation of anti-NTM therapy having less severe disease and therefore being observed for a longer duration based on shared decision-making between clinicians and patients [36]. This observation is consistent with our previous findings from a hospital-based NTM-LD cohort, in which a longer interval between microbiological diagnosis and treatment initiation was associated with a lower risk of three-year mortality (aHR = 0.998; *P* = 0.015) [33]. However, the inverse effect was modest, with each additional day before initiation of anti-NTM treatment associated with only an approximately 0.1% change in the risk of three-year mortality.

Several findings warrant cautious interpretation. First, the strongest predictors of mortality – chronic respiratory failure, cancer, heart failure, COPD, and idiopathic pulmonary fibrosis – are established markers of frailty, rather than NTM-specific prognostic factors. This observation suggests that mortality in treated NTM-LD is driven largely by underlying comorbidity burden and competing causes of death, with cancer, chronic airway diseases, and pneumonia being the leading recorded causes of death. Second, the NHIRD does not distinguish NTM species, precluding species-specific analyses. Because treatment response and prognosis differ substantially among species, particularly for *Mycobacterium abscessus*, our estimates of treatment duration, prescribing patterns, and mortality should be interpreted as a population-level, rather than species-specific findings.

The observed increase in NTM-LD incidence should also be interpreted with care. Improved clinician awareness, expanded access to computed tomography of the chest, greater use of mycobacterial culture, and population ageing may have contributed to increased case ascertainment over the study period. These factors may have acted alongside a genuine increase in disease burden; however, we cannot determine their relative contributions based on our data. Nevertheless, the increasing number of patients requiring prolonged multidrug therapy, the concentration of care in tertiary and regional hospitals, and a three-year mortality approaching 20% underscore the growing healthcare burden of NTM-LD and the need for improved surveillance, standardised diagnosis, and multidisciplinary management.

### Limitations

This study has several limitations. First, the NHIRD does not record microbiologic data, so NTM species could not be distinguished using ICD codes. We, therefore, defined the appropriateness of therapy at the regimen level, rather than by species-specific drug susceptibility. Although previous research has identified *Mycobacterium avium* complex and *Mycobacterium abscessus *group as the most common causative organisms of NTM-LD in Taiwan [14], species-level distribution could not be confirmed in our cohort. Further, we defined NTM-LD from administrative diagnostic and prescription claims that were not validated against the combined microbiologic, radiographic, and clinical criteria specified in the relevant guidelines [10]. Administrative coding alone is recognised as insufficient for diagnosing NTM-LD, and the absence of code validation against a clinical reference standard introduces a non-trivial risk of disease misclassification that may compromise internal validity, which might have affected our results in an undeterminable way. To improve specificity, we required treatment-based inclusion, but this introduces a distinct selection problem, as patients receiving multidrug therapy are likely to represent more severe or actively managed disease, while individuals with indolent disease, surveillance-only management, or incomplete microbiologic confirmation are systematically excluded. Second, treatment protocols were not standardised in the database we used for our retrospective study, and because untreated cases could only be identified through diagnostic claims, their true number may be underestimated. Third, the use of population-based data from Taiwan may limit the generalisability of the findings to other countries with different healthcare systems and patient demographics. Fourth, the NHIRD did not contain chest imaging data, so we could not determine whether patients had cavitary lesions, nor did it have data on clinical decision-making, patient behaviour, and smoking status. Finally, although we measured survival from treatment initiation to mitigate immortal time bias, residual immortal time and confounding-by-indication effects may persist, particularly for the associations involving treatment duration and the diagnosis-to-treatment interval. Taken together, the treated cohort should not be taken as representative of all patients with NTM-LD, and our incidence, treatment, and mortality estimates should be generalised only to treated, claims-defined disease.

## CONCLUSIONS

In this population-based study of real-world NTM-LD treatment in Taiwan, we observed a consistent upward trend in the number of both treated and untreated cases. The three-year mortality rate among treated patients was 19.4%; among patients who died, cancer, chronic airway diseases, and pneumonia were the three most common recorded causes of death. Being older, being male, having cardiorespiratory and malignant comorbidities, being hospitalised, and receiving IV antibiotic treatment were factors independently associated with mortality, most likely reflecting underlying host vulnerability, rather than NTM-specific severity.

## Additional material


Online Supplementary Document

## Data Availability

**Data availability:** The data that support the findings of this study are available from Taiwan’s NHIRD. Restrictions apply to the availability of these data, which were used under license for this study.
