# Supplementary material for: Epidemiologic trends and mortality in nontuberculous mycobacterial lung disease: a nationwide cohort study in Taiwan (2011–2020)
Source: J Glob Health. 2026 Sep 11;16:04292. doi: 10.7189/jogh.16.04292 (PMC13564893; doi:10.7189/jogh.16.04292)
Supplement: Supplementary file 1 [file jogh-16-04292-s001.pdf]

Supplement to: Chang C, Wei Y, Wang P, Pan S, Wang S, Chang C, Shu C. Epidemiologic trends and mortality in non tuberculous mycobacterial lung disease: a nationwide cohort study in Taiwan (2011–2020). J Glob Health. 2026;16:04292.

STROBE Statement—checklist of items that should be included in reports of observational studies

Study: Epidemiologic Trends and Mortality in Nontuberculous Mycobacterial Lung Disease: A Nationwide Cohort Study in Taiwan (2011–2020). Page numbers refer to the final manuscript (NTM-LD_JoGH_FINAL, 0701 version), as paginated when exported to PDF.

	Item No.	Recommendation	Page No.	Relevant text from manuscript
**Title and abstract**	1	(*a*) Indicate the study's design with a commonly used term in the title or the abstract	1, 3	Title: "A Nationwide Cohort Study in Taiwan (2011–2020)"; Abstract Methods: "In this retrospective cohort study using Taiwan's National Health Insurance Research Database…"
		(*b*) Provide in the abstract an informative and balanced summary of what was done and what was found	2–3	Structured abstract (Background, Methods, Results, Conclusions) reporting the cohort, case definition, primary outcome, key adjusted hazard ratios, and a cautious, non-causal conclusion that explicitly notes cases were defined without microbiologic or radiographic confirmation and untreated individuals were excluded.
**Introduction**				
**Background/rationale**	2	Explain the scientific background and rationale for the investigation being reported	5	"The global incidence and prevalence of NTM-LD have risen significantly over the past two decades … Despite the growing clinical importance of NTM-LD, population-based data on treatment patterns, mortality, and prognostic factors remain limited, particularly in Asia."
**Objectives**	3	State specific objectives, including any prespecified hypotheses	6	"…we aimed to (i) describe nationwide trends in treatment initiation and incidence between 2011 and 2020, (ii) characterize real-world treatment courses and regimen heterogeneity, (iii) quantify three-year all-cause and cause-specific mortality, and (iv) identify clinical factors independently associated with three-year mortality."
**Methods**				
**Study design**	4	Present key elements of study design early in the paper	1–3, 6	"This retrospective cohort study used Taiwan's National Health Insurance Research Database" (Title/Abstract); restated at the start of Methods ("Data Source and Study Population").
**Setting**	5	Describe the setting, locations, and relevant dates,	6–7	"Data were obtained from the

	Item No.	Recommendation	Page No.	Relevant text from manuscript
		including periods of recruitment, exposure, follow-up, and data collection		National Health Insurance Research Database (NHIRD) … We identified patients diagnosed with NTM-LD … between July 2011 and December 2020.” Follow-up for 3-year mortality with administrative censoring at 31 December 2020 (Statistical Analysis).
**Participants**	6	(*a*) *Cohort study*—Give the eligibility criteria, and the sources and methods of selection of participants. Describe methods of follow-up	7	“Inclusion required at least two outpatient or emergency-department visits with NTM infection among the top three diagnoses, or at least one hospitalization with NTM infection among the top five diagnoses. Patients were excluded if they had pulmonary tuberculosis within 6 months … or if they did not receive NTM treatment or received treatment for less than 28 days” (Figure 1).
		(*b*) *Cohort study*—For matched studies, give matching criteria and number of exposed and unexposed	N/A	Not applicable; this is an unmatched cohort study.
**Variables**	7	Clearly define all outcomes, exposures, predictors, potential confounders, and effect modifiers. Give diagnostic criteria, if applicable	8–9	“Covariates included sex, age, care setting, comorbidities, and medications … The primary outcome was 3-year all-cause mortality” (Main Outcome and Covariates). Anti-NTM regimen and treatment course defined explicitly at the regimen level (Data Source and Study Population, page 7–8).
**Data sources/ measurement**	8*	For each variable of interest, give sources of data and details of methods of assessment (measurement). Describe comparability of assessment methods if there is more than one group	7, 9	NTM-LD identified via ICD-9-CM/ICD-10-CM codes (page 7); “Comorbidities were identified using diagnostic codes and catastrophic illness registrations in the NHIRD … Cause-of-death data were obtained from Taiwan's Ministry of Health and Welfare death registry” (page 9). A single, uniform claims-based source was used for all participants.
**Bias**	9	Describe any efforts to address potential sources of bias	9–10	“Defining cohort entry at treatment initiation, rather than at diagnosis, was intended to reduce immortal-time bias arising from the

	Item No.	Recommendation	Page No.	Relevant text from manuscript
				interval between diagnosis and the start of therapy" (Statistical Analysis). Misclassification and selection bias from the claims-based case definition are addressed in the Discussion/Limitations.
**Study size**	10	Explain how the study size was arrived at	7, 11	The study included all eligible patients in the nationwide NHIRD meeting the case definition during 2011–2020 (no a priori sample-size calculation); the final analytic cohort comprised 3,762 treated patients, as detailed in the Figure 1 flow diagram and restated in the Results ("the final cohort comprised 3762 patients with adequate treatment").
**Quantitative variables**	11	Explain how quantitative variables were handled in the analyses. If applicable, describe which groupings were chosen and why	8–9	"Covariates were selected a priori on the basis of clinical and biological plausibility and their established prognostic relevance in NTM-LD … rather than on the basis of statistical significance" (Main Outcome and Covariates). Treatment duration and diagnosis-to-treatment interval modeled as continuous (per-day) variables; age dichotomized at ≥65 years (Table 4).
**Statistical methods**	12	(*a*) Describe all statistical methods, including those used to control for confounding	9–10	"Cox proportional hazards regression was employed to calculate multivariable hazard ratios (HRs) and 95% confidence intervals (CIs) … All covariates were specified a priori … and were entered simultaneously into the multivariable model." Chi-square, Student's t-test, and ANOVA used for descriptive comparisons; SAS v9.4.
		(*b*) Describe any methods used to examine subgroups and interactions	13	No formal subgroup or interaction terms were prespecified in the statistical methods; absolute 3-year mortality is reported descriptively across clinical subgroups (e.g., age, sex, comorbidities, care setting) in the Results.
		(*c*) Explain how missing data were addressed	—	Not explicitly addressed in the text. The NHIRD

	Item No.	Recommendation	Page No.	Relevant text from manuscript
				provides complete administrative ascertainment of coded diagnoses, prescriptions, and vital status for all enrollees, so no imputation was required for the analyzed variables. Variables not captured by the database (e.g., BMI, smoking) are noted as a limitation (page 19–20).
		(*d*) *Cohort study*—If applicable, explain how loss to follow-up was addressed	9–10	"Survival time was measured from the date of treatment initiation … to death or the end of the 3-year follow-up window, with administrative censoring at the database cut-off of 31 December 2020" (Statistical Analysis), minimizing loss to follow-up.
		(*e*) Describe any sensitivity analyses	10	"The proportional hazards assumption was evaluated using scaled Schoenfeld residuals; no material violation was observed." For patients with more than one treatment course, total duration was summed across courses and the number of courses modeled as a separate covariate.
**Results**				
**Participants**	13*	(*a*) Report numbers of individuals at each stage—eg numbers potentially eligible, examined for eligibility, confirmed eligible, included, completing follow-up, and analysed	10–11	"Between July 2011 and December 2020, 21 525 patients were diagnosed with NTM-LD. Of these, 5936 were excluded … leaving 15 589 eligible patients. Among them, 9156 received no anti-NTM treatment and 2671 received treatment for less than 28 days; the final cohort comprised 3762 patients with adequate treatment."
		(*b*) Give reasons for non-participation at each stage	7, 10–11	Exclusions quantified in-text and in the Figure 1 flow diagram: tuberculosis within 6 months (n = 5936); no anti-NTM treatment (n = 9156); treatment <28 days (n = 2671).
		(*c*) Consider use of a flow diagram	7 (cited); Fig. legend p. 31	Figure 1. Study population flow diagram (referenced in Methods, page 7; legend on page 31).
**Descriptive data**	14*	(*a*) Give characteristics of study participants and information on exposures and potential confounders	11, Table 2	"The mean age of the treated cohort was 60.4 years (SD =

	Item No.	Recommendation	Page No.	Relevant text from manuscript
			(pp. 26–28)	15.3) and 50.4% were male … Most patients (2402; 64.2%) received care at medical centers … The most common comorbidities were COPD (24.9%) and bronchiectasis (23.1%)."
		(*b*) Indicate number of participants with missing data for each variable of interest	—	Not explicitly reported; administrative variables were complete for all participants, so no variable analyzed required exclusion or imputation for missingness.
		(*c*) *Cohort study*—Summarise follow-up time	12	"The median follow-up was 1053.5 days …"
**Outcome data**	15*	(*Cohort study*) Report numbers of outcome events or summary measures over time	12, Table 2	"…the three-year mortality rate among treated patients was 19.4%. The leading causes of death were cancer (239 patients; 32.8%), chronic airway diseases (95; 13.9%), and pneumonia (72; 9.9%). A total of 729 patients died during follow-up."
**Main results**	16	(*a*) Give unadjusted and, if applicable, confounder-adjusted estimates and their precision (eg, 95% CI). Make clear which confounders were adjusted for and why	12–13, Table 4 (p. 29–30)	Crude and adjusted hazard ratios with 95% CIs for all covariates reported in Table 4 and the Results (e.g., hospitalization aHR = 3.70, 95% CI = 3.07–4.46; cancer aHR = 3.45, 95% CI = 2.94–4.05). Covariates were prespecified on clinical grounds (Main Outcome and Covariates, page 8–9).
		(*b*) Report category boundaries when continuous variables were categorized	Table 4 (p. 29–30)	Age categorized at ≥65 vs. <65 years; treatment duration and diagnosis-to-treatment interval retained as continuous per-day variables (Table 4).
		(*c*) If relevant, consider translating estimates of relative risk into absolute risk	12–13, 18	"…a longer total treatment duration was associated with lower mortality (aHR = 0.997 per day …), and the interval between diagnosis and treatment initiation showed a statistically significant but clinically negligible association (aHR = 0.999 per day …)" (Results, page 12–13); explicitly translated to absolute terms in the Discussion: "…each additional day before initiation of anti-NTM treatment associated with only an approximately 0.1% change in the risk of 3-year

	Item No.	Recommendation	Page No.	Relevant text from manuscript
				mortality" (page 18).
**Other analyses**	17	Report other analyses done—eg analyses of subgroups and interactions, and sensitivity analyses	12–13	Absolute 3-year mortality reported across clinical subgroups (e.g., chronic respiratory failure 73.8%, cancer 45.2%, hospitalization 35.3%, age ≥65 vs. <65 years 29.7% vs. 11.9%, male vs. female 24.4% vs. 14.2%) and by care setting (regional hospital 23.7%, medical center 17.9%, district hospital 17.4%); proportional-hazards diagnostics performed (Statistical Analysis, page 10).
**Discussion**				
**Key results**	18	Summarise key results with reference to study objectives	13–14	"To our knowledge, this is the first population-based study evaluating the incidence of NTM-LD in Taiwan … In the present study, NTM-LD cases with anti-NTM treatment accounted for approximately one-fourth of all NTM-LD cases, with the incidence increasing from 1.3 to 2.2 per 100,000 population." Treatment patterns, mortality, and associated factors are then summarized against the four study objectives.
**Limitations**	19	Discuss limitations of the study, taking into account sources of potential bias or imprecision. Discuss both direction and magnitude of any potential bias	19–21	"This study has several limitations. First, the NHIRD does not record microbiologic data … NTM-LD was defined from administrative diagnostic and prescription claims that were not validated against the combined microbiologic, radiographic, and clinical criteria … the direction of any resulting bias cannot be determined … treatment-based inclusion … introduces a distinct selection problem … residual immortal-time and confounding-by-indication effects may persist."
**Interpretation**	20	Give a cautious overall interpretation of results considering objectives, limitations, multiplicity of analyses, results from similar studies, and other relevant evidence	18–19	"Several findings merit further interpretation. First, the strongest predictors of mortality … are established markers of frailty and limited physiological reserve rather than NTM-specific prognostic factors … The

	Item No.	Recommendation	Page No.	Relevant text from manuscript
				observed increase in NTM-LD incidence should also be interpreted cautiously … likely contributed to increased case ascertainment in addition to any true rise in disease burden.”
**Generalisability**	21	Discuss the generalisability (external validity) of the study results	21	“The use of population-based data from Taiwan may limit the generalizability of the findings to other countries with different healthcare systems and patient demographics … Taken together, the treated cohort does not represent all patients with NTM-LD, and our incidence, treatment, and mortality estimates should be generalized only to treated, claims-defined disease.”
**Other information**				
**Funding**	22	Give the source of funding and the role of the funders for the present study and, if applicable, for the original study on which the present article is based	23	“This study was partially supported by a research grant from National Taiwan University Hospital (No. 113-E0007, 114-E0007, and 115-E0007). The funders had no role in study design, data collection and analysis, decision to publish, or preparation of the manuscript.”

**Give information separately for exposed and unexposed groups in cohort and cross-sectional studies, and for cases and controls in case-control studies.*

Note: An Explanation and Elaboration article discusses each checklist item and gives methodological background and published examples of transparent reporting. The STROBE checklist is best used in conjunction with this article (freely available on the Web sites of PLoS Medicine, Annals of Internal Medicine, and Epidemiology). Information on the STROBE Initiative is available at www.strobe-statement.org.

Table S1. Univariable and multivariable Cox proportional hazard regression on the risk factors of NTM-LD 3-years all-cause mortality*

	**Crude HR (95% CI)**	***P*-value**	**Adjusted HR (95% CI)**	***P*-value**
**Age ≥65 years**	2.83 (2.43–3.30)	<0.001	2.18 (1.85–2.58)	<0.001
**Male sex**	1.82 (1.57–2.12)	<0.001	1.40 (1.19–1.65)	<0.001
**Care setting**				
Medical center *vs*. district hospital or lower	0.98 (0.75–1.29)	0.902		
Regional hospital *vs*. district hospital or lower	1.38 (1.03–1.84)	0.029	1.26 (0.93–1.71)	0.128
Chronic respiratory failure	7.71 (6.32–9.40)	<0.001	3.22 (2.56–4.04)	<0.001
Cancer	3.93 (3.38–4.58)	<0.001	3.45 (2.94–4.05)	<0.001
Cirrhosis	4.20 (1.74–10.12)	0.001	2.61 (0.96–7.06)	0.059
End-stage renal disease	2.23 (1.64–3.03)	<0.001	1.35 (0.97–1.89)	0.079
Heart failure	3.08 (2.47–3.85)	<0.001	1.59 (1.25–2.01)	<0.001
Diabetes mellitus	1.76 (1.49–2.08)	<0.001	1.02 (0.85–1.22)	0.828
Asthma	1.35 (1.08–1.68)	0.008	0.91 (0.72–1.15)	0.434
COPD	2.17 (1.87–2.52)	<0.001	1.51 (1.27–1.80)	<0.001
Bronchiectasis	0.60 (0.49–0.73)	<0.001	0.85 (0.68–1.06)	0.139
Idiopathic pulmonary fibrosis	2.35 (1.80–3.07)	<0.001	2.54 (1.90–3.39)	<0.001
Gastroesophageal reflux disease	1.16 (0.95–1.42)	0.145		
Sinusitis	1.15 (0.72–1.84)	0.550		
Post-transplant	2.08 (1.27–3.42)	0.004	1.48 (0.89–2.47)	0.129
Autoimmune disease	0.96 (0.65–1.41)	0.832		
Sjögren syndrome	0.69 (0.44–1.07)	0.097		
Hospitalization	5.21 (4.38–6.19)	<0.001	3.70 (3.07–4.46)	<0.001
Total treatment duration, per day	0.996 (0.996–0.997)	<0.001	0.997 (0.995–0.9998)	0.036
First-course duration, per day	0.997 (0.996–0.997)	<0.001	0.999 (0.997–1.002)	0.630
Diagnosis-to-treatment interval, per day	0.999 (0.999–1.000)	0.034	0.999 (0.998–0.999)	<0.001
≥2 *vs*. 1 treatment course	0.44 (0.31–0.62)	<0.001	0.64 (0.38–1.07)	0.089
Treated with IV antibiotics	2.14 (1.80–2.54)	<0.001	1.46 (1.19–1.78)	<0.001
Treated with IV amikacin	1.27 (0.99–1.62)	0.059		

CI – confidence interval, COPD – chronic obstructive pulmonary disease, HR – hazard ratio, IV – intravenous, NTM-LD – nontuberculous mycobacterial lung disease

*Blank multivariable cells indicate variables not retained in the multivariable model.
